# Metabolic characteristics of intracellular trehalose enrichment in salt-tolerant *Zygosaccharomyces rouxii*

**DOI:** 10.3389/fmicb.2022.935756

**Published:** 2022-08-02

**Authors:** Yangjian Wei, Zhenzhen Yan, Mengqi Liu, Dunwu Chen, Xiong Chen, Xin Li

**Affiliations:** Hubei Collaborative Innovation Center for Industrial Fermentation, Key Laboratory of Fermentation Engineering (Ministry of Education), Hubei Key Laboratory of Industrial Microbiology, Hubei University of Technology, Wuhan, China

**Keywords:** *Zygosaccharomyces rouxii*, metabolic characteristics, carbohydrate, amino acids, organic acids, long-term temperature pressure

## Abstract

The salt-tolerant flavor yeast *Zygosaccharomyces rouxii* is an important food flavor microorganism, but its intracellular stress-resistant trehalose synthesis efficiency has been shown to be low, resulting in its weak high-temperature resistance. The intracellular and extracellular levels of carbohydrates, organic acids, and amino acids of *Z. rouxii* in a 20-L mechanically stirred ventilated fermenter were analyzed using metabolomics research methods. Our results showed that glucose supplementation could promote the growth of yeast cells, but high temperatures (> 35°C) significantly prevented cell growth. Under three different growth strategies, extracellular glucose was continuously utilized and intracellular glucose was continuously metabolized, but glucose overflow metabolism was inhibited by high temperature, which showed that the level of intracellular/extracellular ethanol was stable. High temperature stimulated significant intracellular trehalose accumulation (*c*_20_._5*h*_ = 80.78 mg/g Dry Cell Weight (DCW)) but not efflux, as well as xylitol accumulation (*c*_20_._5*h*_ = 185.97 mg/g DCW) but with efflux (c_20_._5*h*_ = 29.78 g/L). Moreover, heat resistance evaluation showed that xylitol and trehalose had heat-protective effects on *Z. rouxii*. In addition, a large amount of propionic acid and butyric acid accumulated inside and outside these cells, showing that the conversion of glucose to acid in yeast cells becomes the main pathway of glucose overflow metabolism in high temperatures. In addition, the increased demand of yeast cells for phenylalanine, threonine, and glycine at high temperatures suggested that these metabolites participated in the temperature adaptation of *Z. rouxii* in different ways. Valine and leucine/isoleucine [branched-chain amino acids (BCAAs)] were mainly affected by the addition of glucose, while glucose, sucrose, aspartic acid/asparagine, and glutamate/glutamine were not affected by this temperature regulation as a whole. This study could help deepen our understanding of the high-temperature adaptation mechanism of salt-tolerant *Z. rouxii*, and has theoretical significance for the application of highly tolerant yeast to food brewing.

## Introduction

*Zygosaccharomyces rouxii* (*Z. rouxii*) is one of the main fermentation yeasts used in soy sauce brewing (Solieri, 2021). A large number of esters and polyols are generated during this fermentation process, and the levels of succinic acid and furfural in soy sauce brewing can be high, both of which play a vital role in the production of soy sauce flavor (Guo et al., 2016). Studies have shown that in the process of soy sauce fermentation using *Z. rouxii*, a high salt environment results in the accumulation of a large amount of Na^+^ in these cells, affecting ion balance and producing osmotic stress. At the same time, the nutritional limitations of late fermentation force cells to enter a stable growth phase, slowing down their metabolism, and even stopping fermentation (Solieri et al., 2016; Lei et al., 2019). These environments greatly impact yeast cell activity and transformation ability (Andreu et al., 2022), and in particular, stress resistance has been crucial for the development of yeast fermentation capabilities in the biological industry.

The potential mechanism of yeast tolerance to an environment has been shown to be very complex and often includes many genes, proteins, and metabolites (Flores-Cotera et al., 2021). Pereira et al. (2018) found that during the heat stress reaction in a W303-1A *Saccharomyces cerevisiae (S. cerevisiae)* strain, ATP and NADPH-dependent enzyme genes were significantly upregulated, and the production of protective molecules (trehalose, glycerol, and heat shock proteins) increased sharply during the first 10 min of heat shock, with the resources allocated to each glycolysis flux branch reaching a balance at 10 min until 20 min after heat stress stabilization (Pereira et al., 2018). In addition, Chen et al. (2016) used an *S. cerevisiae* S288c strain for studying ethanol fermentation. In the process of stagnation-exponential phase transition, yeast cells adapt to these pressures, and the Tricarboxylic Acid Cycle (TCA) cycle was shown to be downregulated. During the exponential-stable phase transition, reactivation of the TCA cycle provides sufficient energy, while the levels of glycerol, trehalose, ergosterol, and some amino acids increased, jointly giving yeast cells higher ethanol tolerance (Chen et al., 2016). As a complex living system, yeast adaptation or tolerance to environmental stress was shown to be related not only to the cell membrane or single organelles but also to the whole system (Saini et al., 2018). Therefore, it is crucial to understand how yeast cells respond to changing environments at the system level during continuous fermentation.

It should be noted that continuously fed glucose can be used as a molecular signal to promote the glycolysis pathway, and elevated temperature can stimulate the stress metabolic response of yeast cells. However, the treatment of yeast cells with short-term stresses only represents the instantaneous response characteristics of these cells. The industrial fermentation process is often accompanied by a slow-changing environment. In this study, based on metabolomics research methods, the metabolic characteristics of *Z. rouxii* during feeding and in heat shock-coupling conditions were analyzed, which has unique theoretical significance for the application of highly tolerant yeasts in the food industry.

## Materials and methods

### Strains and medium

*Zygosaccharomyces rouxii* isolated from the Chinese traditional food brewing by the functional yeast and brewing microorganism laboratory was used in this research and stored in the Chinese typical culture preservation center (CTCC M 2013310).

Yeast extract peptone dextrose medium (YEPD) (glucose, 2%; yeast extract, 1%; and peptone, 2%) was used as a preservation medium, and a live cell-counting medium was YEPD with 1.5% agar added.

Optimized molasses medium was used as a seed and fermentation medium and consisted of molasses, 38%; yeast powder, 0.5%; KH_2_PO_4_, 1.0%; KCl, 0.05%; (NH_4_)_2_SO_4_, 0.5%; and MgSO_4_^.^7H_2_O, 0.06%.

### Basic fermentation conditions

The *Z. rouxii* strain used in this study was precultured in YEPD broth for 24 h and then inoculated into an optimized molasses medium at 30°C and shaken at 200 r/min in 250 ml cotton-plugged flasks. The inoculum dose was adjusted to keep the OD_600_ of the initial inoculated fermentation culture at 0.1.

Trehalose fermentation was completed in a 20-L mechanical stirring ventilation fermenter. The basic fermentation conditions were as follows: the initial fermentation medium volume was 7.2 L, the fermentation temperature was set to 30°C using the non-control interval, the initial setting speed was 450 r/min, and the aeration rate was 1.3 air volume/culture volume/min (VVM). The pH value of the fermentation medium was maintained at 5.0 using 3 M NaOH and 3 M HCl. A 0.5% v/v antifoaming agent was used to prevent the accumulation of foam during the fermentation process.

### Fermentation regulation strategy

The different fermentation control strategies used in this study are shown in Table 1. Strategy 1 was a non-regulation fermentation process, which was used as a basic control. Strategy 2 was a feeding-only control process and Strategy 3 was a control strategy coupling feeding and temperature control. In addition, glucose was used for feeding regulation at a concentration of 800 g/L.

**TABLE 1:** Three fermentation control strategies.

Fermentation strategies	Feeding	Temperature
Control	–	–
1	8–12 h, 80 mL/h; 12–20 h, 200 mL/h	–
2	8–12 h, 80 mL/h; 12–20 h, 200 mL/h	12–14 h, 35°C; 14–16 h, 37°C, 16–18 h, 39°C; 18–19 h, 41°C; 19–20 h, 43°C (5 gradient)

80 mL/h and 200 mL/h are defined as low-speed feeding and rapid feeding, respectively. Mild temperature stress was considered for all temperatures below 39°C, and serious temperature stress was considered for those above 39°C (including).

### Sample handling

The sampling points were the first and second half-hour of fermentation regulation, and four samples were taken at each time point. Three samples (3 ml fermentation broth) were quenched and used for intracellular metabolite detection (two for testing and one for backup). One sample was directly centrifuged (5 ml fermentation broth) to measure yeast biomass and extracellular metabolites.

### Biomass detection

The concentration of yeast biomass was determined by the measurement of absorbance at 600 nm (Lee et al., 2017). The analyzed biomass samples were centrifuged at 8,000 *g* and −4°C for 5 min, rinsed with a quencher, and then centrifuged again. The obtained pellet was suspended in deionized water and the biomass concentration was measured. Biomass concentration was measured based on a calibration curve prepared from a dried yeast suspension.

### Preliminary treatment of fermentation broth

The fermentation broth used for intracellular metabolite analysis was first treated using a quenching operation (Teng et al., 2008). Briefly, 3 ml of fermentation broth collected from the fermenter was immediately added to 5 ml of precooled −40°C 60% methanol/water (v/v) solution. The liquid was shaken well and centrifuged at 8,000 *g* and −4°C for 5 min. Then, 5 ml of −40°C 60% methanol–water solution was then used to rinse the yeast cells two times. Subsequently, 2 ml of 40% ethanol and glass beads (1/5, w/w for cell wet weight/glass beads) were added to the quenched yeast cell cake and samples were vortex-shaken for 20 min. The mixture was then centrifuged at 8,000 *g* and −4°C for 5 min. Then, the extract for intracellular ethanol detection was replaced with 2 ml of deionized water (wet cell/glass beads 1/5, w/w). All supernatants were transferred to 2 ml centrifuge tubes and centrifuged at 12,000 *g* and −4°C for 5 min. These supernatants were retained for the detection of intracellular metabolites and stored at −80°C until use.

Furthermore, 5 ml of unquenched supernatant (fermentation broth) was first centrifuged at 8,000 *g* and 4°C for 5 min, 1 ml of supernatant was added with an equal volume of ethanol (supernatant/ethanol was 1/1, v/v), and then centrifuged at 12,000 *g* for 5 min. The supernatant (extracellular metabolite solution) was retained for the detection of extracellular carbohydrates and other metabolites. Another 500 μl of centrifugation supernatant was used for ethyl chloroformate (ECF)-derived determination of extracellular amino acids, and 400 μl of centrifuged supernatant was used to determine the levels of extracellular organic acids. All samples were stored at −80°C until use.

### Sample handling before testing

All samples were subjected to a silylation reaction for the detection of intracellular and extracellular carbohydrates (Chen et al., 2016). Then, 400 μl of intracellular metabolite extract or extracellular metabolite solution was dried using an ultralow-temperature freeze. The dried powder was re-dissolved with 200 μl of pyridine for intracellular metabolite detection or 250 μl of pyridine for extracellular metabolite detection. The pyridine solution and 1 mg/ml 2-ketoglutarate (the internal standard, CAS No. 328-50-7) were processed using a silylation reaction in a gas phase bottle, respectively. An equal volume of each solution was first mixed and then centrifuged at 8,000 *g* for 1 min. The supernatant was collected for the detection of extracellular metabolites. The amounts of oximation agent [20 mg/ml methoxyamine hydrochloride (CAS No. 593-56-6); pyridine solution] and silylation agent [*N*-methyl-*N*-(trimethylsilyl) trifluoroacetamide, CAS No. 24589-78-4] in 100 μl of the sample were 50 and 80 μl, respectively. The oximation and silylation reactions were carried out in a 40°C water bath for 80 min.

The samples were processed using an ECF (CAS No. 541-41-3) reaction for the detection of intracellular and extracellular amino acids according to the method of Reddy et al. (2016; Han et al., 2020). Then, 500 μl of intracellular metabolite extract or extracellular metabolite solution was made alkaline by adding 45 μl of 7 M NaOH. Next, 500 μl of a mixture of anhydrous ethanol and pyridine (V:V = 4:1) was then added, and samples were shaken. In addition, 100 μl of ECF was added to each sample, and samples were sonicated two times for 1 min. Finally, 500 μl of a chloroform solution contains 5 μl of ECF and 50 μl of an internal standard (0.2% phenethyl acetate), and 200 μl of a saturated NaHCO_3_ solution was added to each sample; the samples were shaken vigorously for 1 min and centrifuged at 3,000 *g* for 5 min. The lower layer was transferred to a 2-ml centrifuge tube with anhydrous sodium sulfate added, and this was centrifuged at 3,000 *g* for 1 min followed by sample detection.

The preliminary treatment of fermentation broth for the detection of organic acids was performed according to Ulbrich et al. (2014). First of all, 400 μl of intracellular metabolite extract or extracellular metabolite solution was put into a 2-ml centrifuge tube, 1.6 ml of methanol was added, samples were shaken and mixed evenly, and finally centrifuged at 12,000 *g* for 5 min. The supernatant was filtered through a Millipore filter (0.22 μm). Following this, 400 μl of tert-amyl alcohol (0.04%, 65% ethanol as a solvent) (internal standard, CAS No. 75-85-4) and 50 μl of formic acid (activator) were added to 550 μl of filtrate. The mixture was shaken and then stored at −20°C until use.

### Gas chromatographic conditions

All metabolites were detected on a GC7890B (Agilent Technologies Inc., America) with a hydrogen flame ion detector. Nitrogen gas was used as a carrier gas and the flow rate was 5 ml/min. The injection volume was 1 μl.

A DB-5 capillary column (30 m × 250 mm × 0.25 μm, Agilent J&W, Scientific Folsom, CA, United States) was used for the detection of amino acids and sugars. The non-split injection mode was adopted. The column temperature was set to 70°C for 5 min, followed by a 5°C/min rate to 280°C for 5 min. The inlet temperature was set to 280°C, and the detector temperature was set to 300°C.

An HP-5 capillary column (Agilent, 30 m× 0.32 mm× 0.25μm) was employed for the detection of organic acids. The split ratio was 10:1. The inlet temperature and the detector temperature were set to 250 and 280°C, respectively. The column temperature was set to 40°C for 5 min, followed by a 20°C/min rate to 120°C, and a 10°C/min rate to 220°C, which was maintained for 3 min.

The qualitative determination of metabolites was completed by comparing the retention time of substances in standards and samples. The concentration of intracellular metabolites (mg/g Dry Cell Weight (DCW)) is expressed as the mass of metabolites contained in unit biomass. The concentration of extracellular metabolites (g/L) is expressed by the mass of each metabolite per unit volume.

### Thermal protection evaluation

*Zygosaccharomyces rouxii* cells cultured at 30°C and 200 r/min in YEPD liquid medium were used to evaluate the thermal protection of xylitol and trehalose. The heat treatment time was 20 min, and the heat treatment temperature was set at 40, 45, and 50°C. The final concentration of exogenous additives was 2%, but the mass ratio of trehalose and xylitol was 1:1. The cell culture medium of *Z. rouxii* without exogenous substances and heat shock was used as a control. The number of viable cells (CFU/ml) of *Z. rouxii* was measured using the dilution coating plate method. The survival rate was defined as the percentage ratio of the number of living cells after heat treatment to the number of control living cells.

### Statistical methods

All laboratory analyses were performed in triplicate. Statistical analysis (calculation of mean value, determination of SD) was carried out using Excel and the change diagram was drawn using the OriginPro 2019b software (Seifert, 2014). The IBM SPSS Statistics 23.0 software (SPSS Inc., Chicago, IL, United States) was used for determining the significant differences for the analysis of variance.

## Results

### Growth difference analysis

**FIGURE 1:**
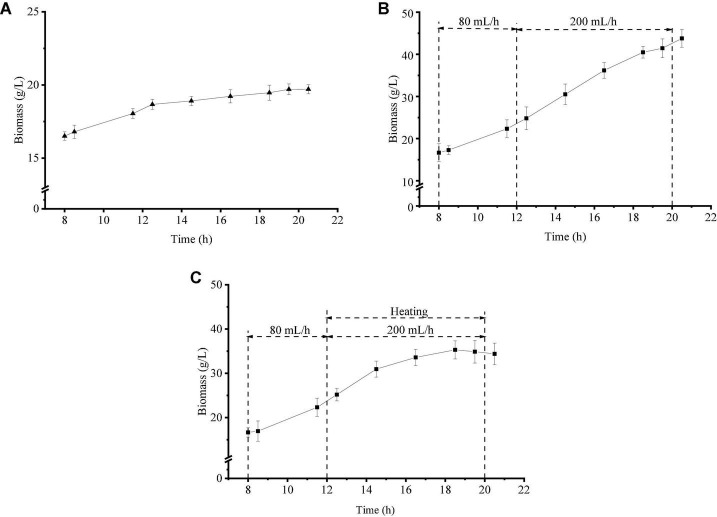
Growth differences of *Z. rouxii* using three fermentation regulation strategies. **(A)** Unregulated, **(B)** strategy 1, **(C)** strategy 2.

Cell biomass was used to evaluate the growth trend of *Z. rouxii* in this study. Under the three growth regimes, the biomass at 8 h was about 16.60 g/L, as shown in Figure 1. The biomass of strategy one (Figure 1A) remained stable as a whole, which showed that the growth stage of yeast cells was the stable stage in this culture condition. The addition of glucose as the only carbon source (Figure 1B) could stimulate the growth of *Z. rouxii* and the maximum biomass reached 43.78 g/L by the end of fermentation. When temperature pressure was applied to this fermentation system (Figure 1C), the biomass growth was divided into two regions, namely, a low-speed growth stage (12–18 h, I phase) and a non-growth stage (18–20 h, II phase). The maximum biomass using strategy 3 was 35.30 g/L at 18.5 h. These results indicated that *Z. rouxii* could tolerate medium-temperature pressure (≤ 39°C) and lost proliferation abilities under high-temperature selective pressures (> 39°C).

### Intracellular and extracellular carbohydrate metabolites

**FIGURE 2:**
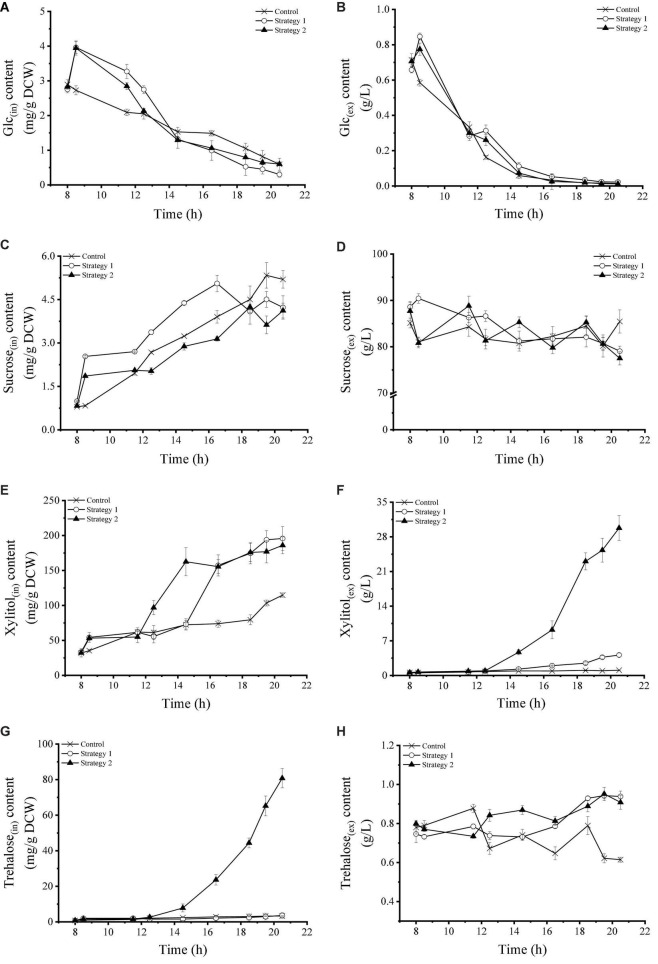
**(A–H)** Changes in the intracellular and extracellular carbohydrates content of *Z. rouxii* using three fermentation regulation strategies. “in” stands for intracellular material, “ex” stands for extracellular material.

Excluding the short-term accumulation caused by the start-up of feeding, the intracellular and extracellular glucose content of *Z. rouxii* yeast cells showed a continuous downward trend using these three growth strategies (Figures 2A–B). In particular, the extracellular glucose content was almost removed by 16 h. This result indicated that the glucose utilization and metabolism of *Z. rouxii* were minimally affected by feeding and temperature regulation. The concentration of intracellular and extracellular glucose did not increase greatly when the growth of yeast cells was prevented and the feeding operation did not stop (18–20 h). This phenomenon implied that the metabolism of glucose was not halted, but its metabolic use had likely changed. The changes in intracellular and extracellular sucrose concentrations of *Z. rouxii* are shown in Figures 2C,D. Under these three fermentation conditions, intracellular sucrose accumulated slowly in cells, and the extracellular sucrose in the fermentation broth decreased slowly, and this was not affected by the addition of glucose or gradient heating. As *Z. rouxii* could not use sucrose as a carbon source, the change in sucrose may have been a by-product of carbohydrate transport.

In a normal environment, the intracellular xylitol content continued to rise slowly and reached a maximum value of 114.80 mg/g DCW by the end of fermentation, while extracellular xylitol did not accumulate. When carbohydrates were added to the fermentation system, the ability of *Z. rouxii* to synthesize xylitol was improved (mainly during the early stage of high-speed feeding). Most xylitol accumulated inside these cells, while a small amount was secreted outside the surrounding medium. When high-speed feeding was combined with gradient heating control (12–20 h in strategy 2), the initial time for yeast cells to accumulate xylitol in the cell cytoplasm was 2 h earlier, and a large amount of xylitol was removed from the cell, resulting in a continuous increase in xylitol content in the fermentation broth (Figures 2E–F). The extracellular xylitol concentration at the fermentation endpoint was 29.78 g/L. Notably, the intracellular xylitol concentration at the fermentation endpoint in strategies 1 (195.73 mg/g DCW) and 2 (185.97 mg/g DCW) was not significant. These results showed that *Z. rouxii* possesses the ability to synthesize xylitol from glucose and the accumulation of intracellular xylitol was closely related to the addition of glucose. Temperature stress, it seemed, stimulated xylitol metabolism earlier and the efflux of xylitol in *Z. rouxii*.

The activation of trehalose anabolism is a strategy used by many organisms to increase the tolerance to adverse environmental conditions, and its accumulation can change significantly depending on growth, nutrient, and stress conditions (Chen et al., 2019). Under normal physiological conditions, the supplement of carbohydrates cannot activate the synthesis of trehalose. However, under the condition of carbon supplementation coupled with high temperature, the intracellular trehalose level of *Z. rouxii* increased significantly, and the maximum concentration reached 117.93 mg/g DCW by the end of fermentation (Figures 2G–H). The stability of extracellular trehalose levels suggested that the transport channel for trehalose was closed at high temperatures. Unlike xylitol, high-temperature stimulation and sufficient carbohydrates played an equally important role in the synthesis of trehalose by *Z. rouxii*.

### Intracellular and extracellular overflow metabolites

Early studies have shown that glycolysis flux increases when energy sources change from energy-efficient respiration to inefficient respiration or overflow metabolism caused by fermentation metabolism (de Groot et al., 2020).

**FIGURE 3:**
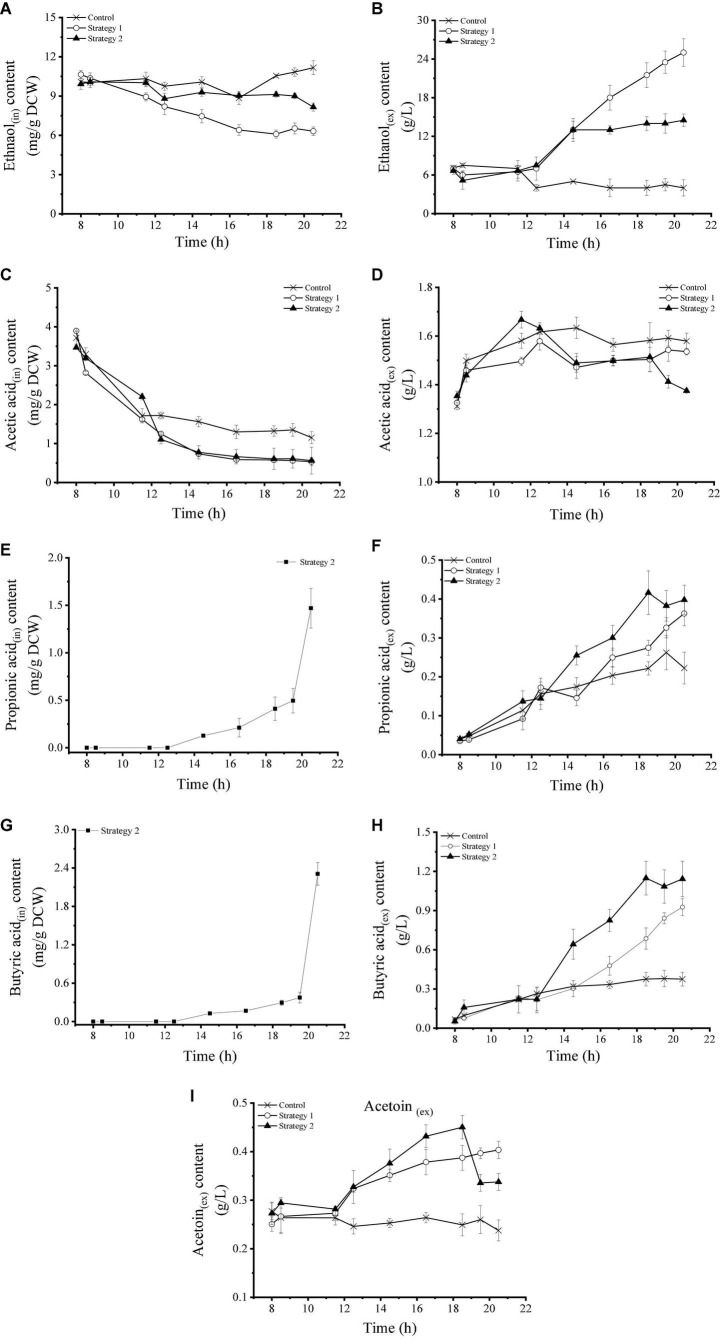
**(A–I)** Changes in intracellular and extracellular overflow metabolite levels in *Z. rouxii* using three fermentation regulation strategies. “in” stands for intracellular material, “ex” stands for extracellular material.

The change in the content of intracellular and extracellular organic acids in *Z. rouxii* using these three fermentation control strategies is shown in Figure 3. Intracellular and extracellular ethanol concentrations remained stable under control conditions. After high-speed glucose supplementation (strategy 2), the intracellular ethanol accumulation concentration decreased, while the extracellular ethanol content increased significantly, and its maximum level reached 25.00 g/L. Under regulation strategy 3, the level of intracellular ethanol was similar to that of the control, but the content of extracellular ethanol was stable at 14.00 g/L after a small amount of accumulation during the early stage of heating. This result indicated that the metabolic pathway of sugar alcohol conversion activated by carbohydrate supplementation was shut off during temperature stress. Compared with ethanol metabolism, feeding and temperature pressure affected acetic acid metabolism less. The changes in intracellular and extracellular acetic acid levels in strategies 2 and 3 were similar, and the concentration levels were lower than those in the control.

Propionic acid and butyric acid were two organic acids that were significantly affected by temperature pressure (Figures 3E–H). Compared with the control, high-speed feeding of carbohydrates significantly increased the levels of extracellular propionic acid and butyric acid. When temperature stress was added, the intracellular concentrations of these two organic acids were further increased. Unexpectedly, *Z. rouxii* cells only accumulated propionic acid and butyric acid under high-temperature stress, and the maximum intracellular concentrations reached 1.47 mg/g DCW and 2.31 mg/g DCW by the end of fermentation, respectively. Intracellular acetoin in *Z. rouxii* was not detected, and the changing trend of extracellular acetoin was similar to that of propionic acid and butyric acid. The addition of glucose (strategy 1) significantly increased the content of extracellular acetoin. Under the condition of temperature pressure (strategy 2), the growth trend was more obvious in the mild pressure stage, and the level of acetoin reached a maximum of 0.45 g/L at 18.5 h, but its production was inhibited under serious temperature pressure.

### Intracellular and extracellular amino acids metabolites

**FIGURE 4:**
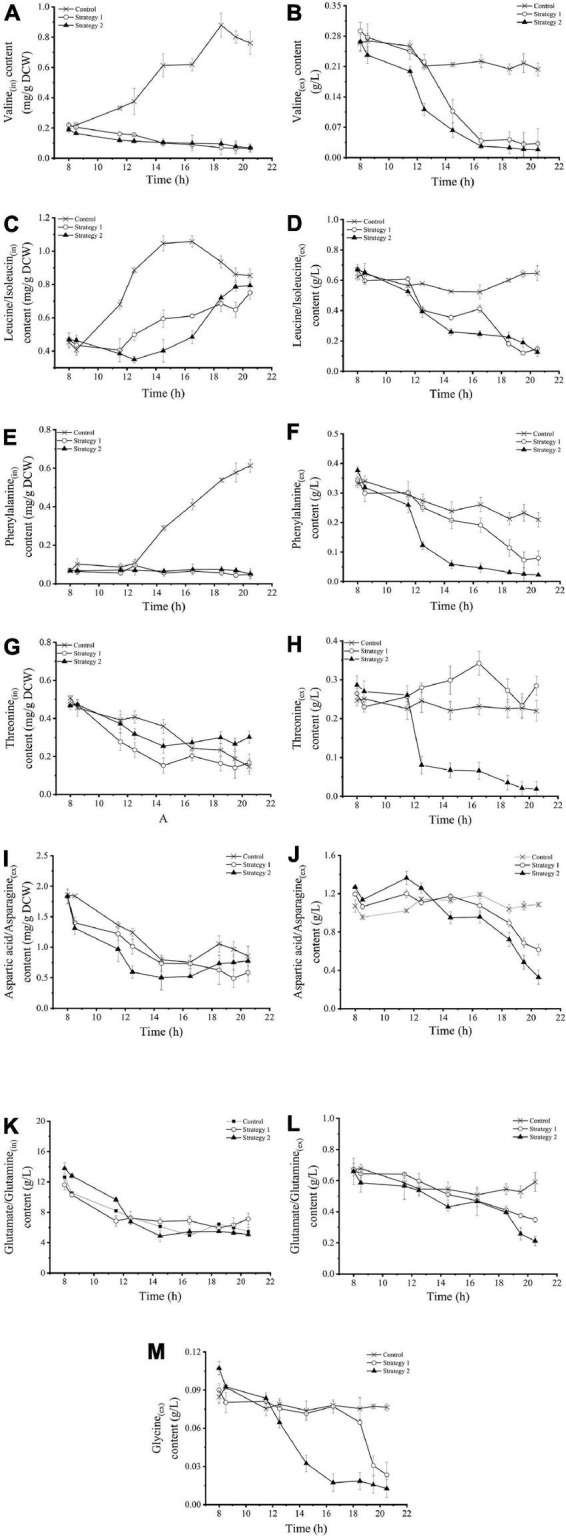
**(A–M)** Changes in intracellular and extracellular amino acid concentrations in *Z. rouxii* using three fermentation regulation strategies. “in” stands for intracellular material, “ex” stands for extracellular material.

The levels of intracellular and extracellular amino acids in *Z. rouxii* grown using these three fermentation control strategies are shown in Figure 4. In the control, the intracellular levels of valine and leucine/isoleucine [branched-chain amino acids (BCAAs), Figures 4A–D] under the regulator stationary phase were increased from a very low level to a maximum level of 0.88 mg/g DCW (18.5 h) and 1.04 mg/g DCW (14.5 h), respectively, and these levels then decreased slightly. At the same time, their extracellular levels were almost stable. However, the responses of BCAAs to fermentation regulation were different. Under regulation strategy 2, the content of extracellular BCAAs decreased significantly, which meant that the addition of carbohydrates strengthened the demand of *Z. rouxii* yeast cells for BCAAs. Temperature pressure only stimulated the faster utilization of exogenous valine, but not leucine/isoleucine, by these yeast cells. The concentration of intracellular valine was consistently maintained at a very low level below 0.1 mg/g DCW. In contrast to this, the intracellular level of leucine/isoleucine gradually increased during the gradient heating stage. These results illustrated that the addition of carbohydrates strengthened the demand for BCAAs (especially valine) in *Z. rouxii*, but high temperature weakened the species’ synthesis ability, resulting in cells using more exogenous amino acids.

The changes in intracellular and extracellular phenylalanine (Figures 4E,F) were very similar to those of valine. Extracellular phenylalanine decreased sharply during the early stage of gradient heating (12–14.5 h), decreasing by 0.20 g/L, which accounted for about 80% of the total change. Carbohydrate supplementation and temperature stress did not change the continuous downward trend of intracellular threonine (Figure 4G), aspartic acid/asparagine (Figure 4I), and glutamate/glutamine (Figure 4K) under normal physiological conditions. However, high-speed feeding resulted in a small amount of extracellular threonine accumulation (Figure 4H), and high temperature resulted in a large decrease in threonine and a small decrease in aspartic acid/asparagine (Figure 4J). Glycine was not detected in the intracellular sample from *Z. rouxii*. The concentration of extracellular glycine (Figure 4M) was stable at 0.8 g/L using the control strategy. In strategy 1, the extracellular glycine concentration decreased sharply during the late stage of fermentation. In the case of high-temperature treatment (strategy 2), the glycine concentration rapidly decreased from 0.08 g/L to 0.02 g/L during the early stage of coupling regulation (12–16.5 h), and then basically stabilized at about 0.01 g/L. Thus, glycine metabolism was more sensitive to temperature and pressure than carbohydrate supplementation.

Based on the differences in regulatory response characteristics, ten amino acids could be roughly divided into two categories. The first type of amino acid was termed the environment-sensitive amino acids, including valine, phenylalanine, threonine, and glycine. For these amino acids, the feeding operation strengthened the utilization of exogenous amino acids, while the high-temperature pressure intensified the utilization degree. The second category of amino acids was termed environment insensitive amino acids and included aspartic acid/asparagine and glutamate/glutamine. These amino acids were not affected by the regulation operation and their utilization trend was determined by the physiological stage. The unique change in leucine/isoleucine was that continuous carbon source supplementation produced short-term (content unchanged) and long-term (intracellular levels increase and extracellular levels decrease) effects; these two amino acids were more sensitive to feeding than to temperature stimulation.

### Evaluation of the thermal protection of trehalose and xylitol

Considering the great changes in xylitol and trehalose concentrations under the coupling control of temperature stress and feeding, the thermal protection ability of these two natural glucose derivatives on yeast cells was evaluated and is shown in Table 2. At 40°C, the number of viable cells in the control group reached 1.01 ± 0.37 × 10^8^ CFU/ml; when trehalose or xylitol was added, this number was 1.77 ± 0.57 × 10^8^ CFU/ml and 1.07 ± 0.13 × 10^8^ CFU/ml, respectively. In addition, the survival rate of cells in the control group without addition was only 65.16%, while the survival rates of only adding trehalose or xylitol were 114.19%, and 69.03%, respectively. The changing trend of 45 and 50°C was consistent with that of 40°C. The data in Table 2 demonstrate that both trehalose and xylitol could play a protective role, and the protective effect of trehalose was better than that of xylitol. Under the condition that trehalose and xylitol were added at the same time, the cell viability at the three temperatures was lower than that of either single addition. Thus, trehalose and xylitol did not have a synergistic protective effect on *Z. rouxii*. Therefore, trehalose and xylitol perform different physiological functions in the process of fermentation regulation.

**TABLE 2:** Thermal protection evaluation.

Additives		Living cells (CFU/mL)			Survival rate (%)		
**Trehalose**	**Xylitol**	**40°C**	**45°C**	**50°C**	**40°C**	**45°C**	**50°C**

–	–	1.01 ± 0.37 × 10^8^	3.49 ± 0.17 × 10^6^	3.88 ± 0.16 × 10^3^	65.16	2.25	0.0025
+	–	1.77 ± 0.57 × 10^8^	8.78 ± 0.17 × 10^6^	2.30 ± 0.5 × 10^4^	114.19***	5.66***	0.0148***
–	+	1.07 ± 0.13 × 10^8^	6.13 ± 0.33 × 10^6^	1.16 ± 0.26 × 10^4^	69.03***	3.95***	0.0075***
+	+	9.50 ± 0.51 × 10^7^	5.47 ± 0.57 × 10^6^	5.68 ± 0.34 × 10^3^	61.29***	3.53***	0.0037***

Living cells under normal physiological temperature (30°C) and without additives were 1.55 ± 0.28 × 10^8^ CFU/mL. CFU represents colony-forming units. Cell viability without additives as control. Significance analysis of the survival rate of under heat treatment and the survival rate of control, “***” (*P* < 0.001).

## Conclusion and discussion

A study on the stress response and mechanism of microorganisms helped deepen our understanding of the interaction between microorganisms and the environment (Kainth et al., 2021) and has also been helpful for the targeted development of artificial high-tolerance microbial strains (Takagi, 2021). As one of the important indicators of environmental parameters, different temperature environments put different selective pressures on microorganisms, leading to unique microorganisms such as thermophilic (Donati et al., 2016) and psychrophilic (Margesin and Miteva, 2011; Yusof et al., 2021). In contrast to *S. cerevisiae*, which initiates large-scale trehalose synthesis under medium-temperature pressure (Walther et al., 2013), the trehalose metabolic pathway of the salt-tolerant yeast *Z. rouxii* was found to be insensitive to temperature (Chen et al., 2017). In the case of its weak trehalose synthesis ability, *Z. rouxii* needed remedial measures to adapt to a high-temperature environment.

The synthesis of xylitol is one such important remedy, which may play two roles. First, when the glycolysis pathway is weakened, the high-speed metabolism of glucose to xylitol provides a stable and sufficient precursor (glucose 6-phosphate) for the slow synthesis of trehalose. Second, it is an important support for the balance of reduction forces. The results of this study lead to a key question: is 6-phosphate a key metabolite for the regulation of trehalose metabolism, the EMP pathway, and xylitol metabolism regulated by glucose 6-phosphate metabolic flow?

Intracellular propionate and butyric acid accumulation indicated that the EMP pathway was not completely shut off. Moreover, they have been shown to be involved in the tolerance response of yeast to high temperatures. This is a common strategy for microorganisms to adapt to adversity by changing cell membrane components to adjust cell membrane fluidity and permeability (Casares et al., 2019). The accumulation of intracellular propionic acid and butyric acid is thus helpful for the adjustment of the cell membrane components in *Z. rouxii* to adapt to high-temperature environments, and their efflux would be a means for *Z. rouxii* to alleviate acid stress. Acetoin production intracellularly accompanies the accumulation of organic acids at low-temperature pressure stages and can neutralize organic acids during fermentation and maintain the balance of NAD + /NADH in yeast cell metabolism (Sun et al., 2012). The valine, leucine/isoleucine, and phenylalanine that were continuously transported into cells did not accumulate in large amounts, indicating that the three were continuously consumed after entering the cell and may be used for protein synthesis during the growth of yeast. After the coupling of gradient heating control, the concentrations of phenylalanine, threonine, and glycine in the fermentation broth decreased significantly, indicating that they may be involved in the stress response of cells.

This study revealed the response of *Z. rouxii* cells in the stable growth phase to long-term high-temperature stress. An interesting question was that when cells in the adaptive phase encounter long-term high-temperature stress, whether they adopt the same strategy from the adaptive phase to adapt to long-term high-temperature stress.

## Data availability statement

The original contributions presented in the study are included in the article/supplementary material, further inquiries can be directed to the corresponding authors.

## Author contributions

YW: data curation, formal analysis, investigation, validation, and writing – original draft. ZY and ML: methodology. DC: idea and methodology. XC: funding acquisition and writing – review and editing. XL: conceptualization, project administration, and supervision. All authors contributed to the article and approved the submitted version.
